# Cerebrotendinous Xanthomatosis: A practice review of pathophysiology, diagnosis, and treatment

**DOI:** 10.3389/fneur.2022.1049850

**Published:** 2022-12-23

**Authors:** Paulo Ribeiro Nóbrega, Anderson Moura Bernardes, Rodrigo Mariano Ribeiro, Sophia Costa Vasconcelos, David Augusto Batista Sá Araújo, Vitor Carneiro de Vasconcelos Gama, Helena Fussiger, Carolina de Figueiredo Santos, Daniel Aguiar Dias, André Luíz Santos Pessoa, Wladimir Bocca Vieira de Rezende Pinto, Jonas Alex Morales Saute, Paulo Victor Sgobbi de Souza, Pedro Braga-Neto

**Affiliations:** ^1^Division of Neurology, Department of Clinical Medicine, Federal University of Ceará, Fortaleza, Brazil; ^2^Neurogenetics Unit, Department of Neurology, University of São Paulo School of Medicine, São Paulo, Brazil; ^3^School of Medicine, Universidade Federação de Estabelecimentos de Ensino Superior em Novo Hamburgo, Novo Hamburgo, Brazil; ^4^Graduate Program in Medicine: Medical Sciences, Universidade Federal do Rio Grande do Sul, Porto Alegre, Brazil; ^5^Pediatric Neurology, Universidade de Fortaleza, Fortaleza, Brazil; ^6^Hospital Infantil Albert Sabin, Fortaleza, Brazil; ^7^Division of Radiology, Federal University of Ceará, Fortaleza, Brazil; ^8^Center of Health Science, Universidade Estadual do Ceará, Fortaleza, Brazil; ^9^Neurometabolic Unit, Division of Neuromuscular Diseases, Department of Neurology and Neurosurgery, Federal University of São Paulo, São Paulo, Brazil; ^10^Medical Genetics Service and Neurology Service, Hospital de Clínicas de Porto Alegre, Porto Alegre, Brazil; ^11^Department of Internal Medicine, Universidade Federal do Rio Grande do Sul, Porto Alegre, Brazil

**Keywords:** Cerebrotendinous Xanthomatosis, lipid storage disease, *CYP27A1*, chenodeoxycholic acid, inherited metabolic disorders, inborn errors of metabolism

## Abstract

Cerebrotendinous Xanthomatosis represents a rare and underdiagnosed inherited neurometabolic disorder due to homozygous or compound heterozygous variants involving the *CYP27A1* gene. This bile acid metabolism disorder represents a key potentially treatable neurogenetic condition due to the wide spectrum of neurological presentations in which it most commonly occurs. Cerebellar ataxia, peripheral neuropathy, spastic paraparesis, epilepsy, parkinsonism, cognitive decline, intellectual disability, and neuropsychiatric disturbances represent some of the most common neurological signs observed in this condition. Despite representing key features to increase diagnostic index suspicion, multisystemic involvement does not represent an obligatory feature and can also be under evaluated during diagnostic work-up. Chenodeoxycholic acid represents a well-known successful therapy for this inherited metabolic disease, however its unavailability in several contexts, high costs and common use in patients at late stages of disease course limit more favorable neurological outcomes for most individuals. This review article aims to discuss and highlight the most recent and updated knowledge regarding clinical, pathophysiological, neuroimaging, genetic and therapeutic aspects related to Cerebrotendinous Xanthomatosis.

## 1. Introduction

Cerebrotendinous Xanthomatosis (CTX) or Cerebral Cholesterinosis (MIM #213700) is a rare autosomal recessive inherited metabolic lipid-storage disorder related to bile acid biosynthesis pathways (1). CTX is caused by bi-allelic pathogenic variants in *CYP27A1* (2q35), which codes sterol 27-hydroxylase, a mitochondrial enzyme of cytochrome P450 oxidase system. Reduction of the activity of this enzyme leads to increased formation and storage of abnormal lipid content in several tissues, especially tendons, lenses and peripheral, and central nervous system (2).

Several hundreds of cases have been reported since the first description in 1937 by Van Bogaert (3). Current data suggest that the disease appears to be substantially underdiagnosed: the incidence in the United States is between 1:72,000 and 1:150,000, and the disease frequency among Sephardim Jews of Morocco has been estimated to be 6 per 70,000 (4, 5). More than 400 individuals with CTX have been reported worldwide (6), with larger groups of affected individuals being reported in the medical literature from Italy, the Netherlands, Germany, Japan, China, Turkey, Israel, and Spain. CTX incidence ranging from 1:134.970 to 1:461.358 in Europeans, 1:263.222 to 1:468.624 in Africans, 1:71,677 to 1:148,914 in Americans, 1:64,267 to 1:64,712 in East Asians and 1:36,072 to 1:75,601 in South Asians (7).

The aim of the present article is to present current evidence on the main clinical, biochemical, radiologic and treatment aspects of CTX.

## 2. Pathophysiology of CTX

CTX is caused by pathogenic variants in *CYP27A1*, which leads to of deficiency in sterol 27-hydroxylase, a mitochondrial enzyme with a key role in cholesterol metabolism and bile acid synthesis pathways. Multiple variants associated with CTX have been identified, including missense, insertion/deletions, splice-site and nonsense variants, and there is no known clear genotype-phenotype correlation (1, 7).

**Figure 1:**
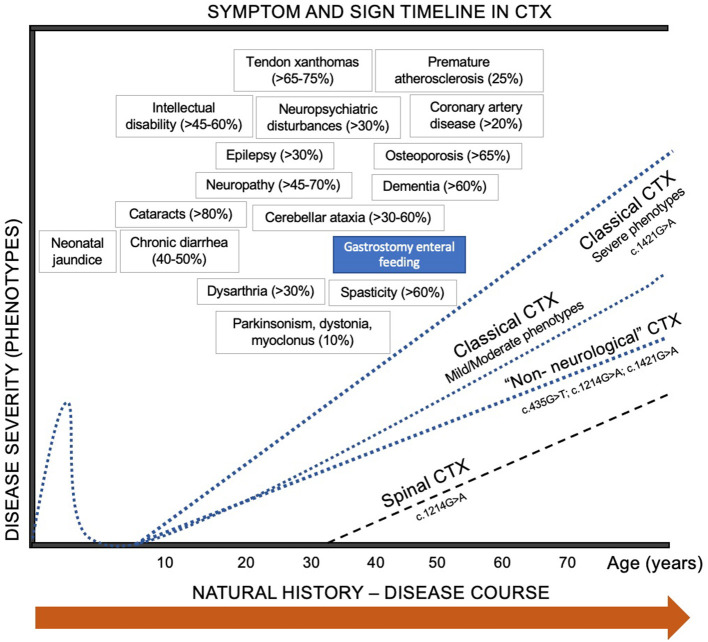
Biochemical pathways of bile acid metabolism and pathophysiological mechanisms involved in CTX. Both alternative (acidic) and classic (neutral) pathways in liver metabolism are represented in blue color. Primary bile acids (Cholic acid and Chenodeoxycholic acid) are represented in orange. Secondary and tertiary bile salts are showed in red. Brain neurometabolic steps are represented in black squares, as well as molecular targets (receptors) of CDCA and CA (1, 9, 10).

Bile acid synthesis occurs in two main metabolic pathways. The classical pathway initiates with 7α-hydroxylation of cholesterol, in which the enzyme cholesterol 7α-hydroxylase acts. The alternative pathway's first step is 27-hydroxylation of cholesterol, catalyzed by sterol 27-hydroxylase, leading to oxidation of side chains of different sterol intermediates (8). In CTX, the impaired activity of *CYP27A1* compromises the formation of chenodeoxycholic acid (CDCA) and cholic acid, to a lesser extent. The loss of the negative feedback effect of CDCA on cholesterol 7α-hydroxylase results in increased levels of 7α-hydroxy-4-cholesten-3-one and its metabolites in the classical pathway (Figure 1). Elevated serum levels of cholesterol and urine bile acids as glucuronides are found (11). The increased cholesterol metabolites adhere to tissues. Furthermore, there are raised levels of other abnormal pathological intermediates, such as cholestanol. Their accumulation mainly in the brain, eye lenses and tendons cause progressive neurologic dysfunction, cataract and xanthomas, respectively, which are some of the classic clinical manifestations of the disease. However, there is a wide number of phenotypes with diverse systemic and neuropsychiatric symptoms (12).

Accumulation of cholestanol in the brain is still not fully understood, since it does not efficiently cross the blood-brain barrier (BBB) (7, 12). Impairment or increased permeability of the BBB has been suggested, possibility endorsed by the high levels of cholestanol and apolipoprotein B found in the cerebrospinal fluid (CSF) in patients with CTX. This change of the BBB may be an effect of circulating bile alcohol glucuronides (1). Nevertheless, some studies have shown an intact BBB in CTX patients, indicating that the increased cholestanol may result from insufficient removal or from synthesis of cholestanol in the brain from cholesterol or another precursor. Furthermore, the bile acid precursor 7α-hydroxy-4-cholesten-3-one crosses the BBB and can be converted to cholestanol by neurons, astrocytes, microglia, and human monocyte-derived macrophages (1, 7) (Figure 1).

The lesions in CTX present significant deposits of cholesterol as well as cholestanol, although serum cholesterol levels are usually normal. However, elevated serum levels of lathosterol and phytosterol are found, indicating increased *de novo* synthesis and incremented intestinal absorption of cholesterol, respectively (11). Also, regarding the metabolism of cholesterol, patients with CTX develop early atherosclerosis and xanthomas, which might be related to diminished transport of peripheral cholesterol to the liver, since the levels of 27-hydroxycholesterol, the product of the 27-hydroxylase activity that passes more efficiently the cell membranes, are significantly reduced (1, 13). Increased cytoplasmic Nε-carboxymethyl-lysine related to oxidative stress dysfunction has been also evidence in foamy histiocytes from the dentate nucleus (14).

## 3. Clinical overview and diagnosis of CTX

**Figure 2:**
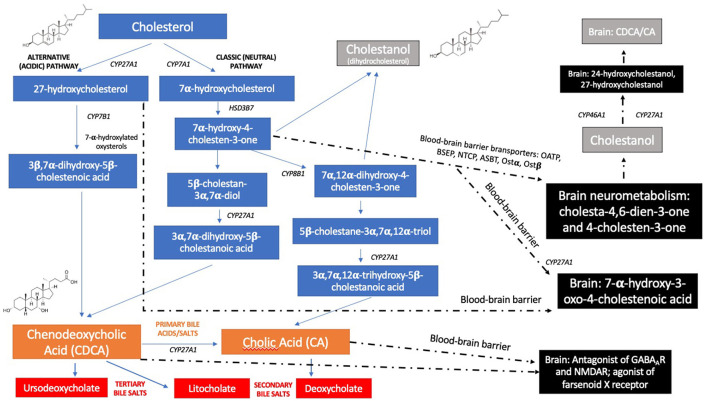
Natural history, disease progression and symptom and sign timeline in each phenotype of CTX (classical, spinal, and “non-neurological” forms), according to the age of symptom-onset (1, 6, 7, 12, 17, 18).

The diagnosis of CTX is mainly based on clinical, neuroradiological, genetic, and biochemical findings. The clinical presentation of the disease is highly heterogeneous, which can lead to significant diagnosis delay. In young people, CTX-related findings are primarily bilateral juvenile cataracts (82%), chronic diarrhea (31%), and intellectual disability (48–74%) (1, 15). In adults, these factors are added to the appearance of tendon xanthomas (76%), as well as psychiatric disturbances (11.4%) and neurological disorders, such as peripheral neuropathy (45%), cerebellar ataxia (36–83%), movement disorders (parkinsonism, dystonia, myoclonus, postural tremor), cognitive decline (87%), and spastic paraparesis and other pyramidal signs (64–92%) (1, 6, 15, 16) (Figure 2).

**Figure 3:**
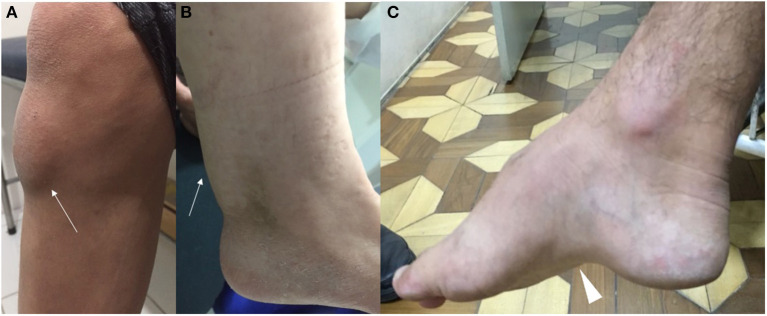
Clinical examination findings in CTX. Note the presence of tendon xanthoma (white arrows) located in the anterior tibial tuberosity **(A)** and the Achilles tendon **(B)**. Pes cavus (white arrowhead) is also another possible feature **(C)**.

In childhood, the earliest manifestations may be infantile diarrhea and neonatal cholestatic jaundice. Typically, jaundice is self-limited and transient, without severe complications, and associated with elevated conjugated hyperbilirubinemia, liver transaminases and alkaline phosphatase. Gamma glutamyl transferase serum levels are generally normal or slightly elevated. The marked reduction of CDCA content does not stimulate the activation and expression of farnesoid X receptor, leading to reduced bile salt exportation and transportation in bile canaliculi (1, 19). There are, however, rare descriptions of severe neonatal cholestasis in patients with CTX, leading to very early lethal progression or evolving with the need of liver transplantation (19). Chronic unexplained infancy-onset diarrhea is the most common gastrointestinal scenario (76% of patients) and occurs due to the presence of bile alcohol in the intraluminal region and relative absence of CDCA. Steatorrhea, cholestanol, cholesterol and fatty acids are absent in the stool content, as well as malabsorption and failure to thrive are not usually observed (1). CDCA represents a highly effective therapy for symptomatic diarrhea remission. Bilateral juvenile cataract is also a common finding, being described in 85% of patients (20). During juvenile and adulthood periods, optic neuropathy and premature retinal vessel atherosclerosis may represent additional neuro-ophthalmological complications (1). Neurological signs and tendinous xanthomas often develop after cataracts appear. Xanthomas may be present in 71% of patients with CTX, appearing in the 1st to 3rd decade of life, being more common in late adolescence and early adulthood. They are represented by large amounts of foamy macrophages full filled with complex lipid crystal cleft structures (1, 7, 12). They appear more frequently in the Achilles tendon but can also be identified in the tibial tuberosity, triceps, and fingers tendons (Figure 3). Tendon involvement in inherited metabolic disorders is not limited to xanthomata in CTX and may also occur in familial hypercholesterolaemia type 3 (*PCSK9*), sitosterolemia (*ABCG8*), ataxia with vitamin E deficiency (*TTPA*), hyperlipoproteinemia type III (*APOE*), primary hypoalphalipoproteinemia type 2 (*APOA1*), Alagille syndrome (*JAG1*), and rarely in congenital hypophosphatasia, ochronosis and galactosemia (21). Other typical examination findings in familial hypercholesterolemia, such as corneal arcus and eyelid xanthelasmata, are not observed in CTX.

Neurological dysfunction is almost always present, with onset usually in late adolescence or early adulthood (12) (Figure 2). Psychiatric symptoms (behavioral disorders, depression, hallucinations, agitation), dementia, and intellectual disability can be present (15). Intellectual disability is commonly one of the most common neurological complications in CTX starting during the first decade of life (12). Pyramidal (spasticity and hyperreflexia) and cerebellar signs (progressive ataxia and dysarthria) are frequent. Although less common, movement disorders, such as parkinsonism, dystonia, myoclonus, and tremor have been reported. Dystonia is mostly multifocal, with reports of blepharospasm, oromandibular, cervical, and limb dystonia (22). Both positive and negative myoclonus have been reported as one of the earlier movement disorders features of CTX, mainly involving upper extremities, which can have a polyminimyoclonus pattern, resembling intention, or action tremor (23). Palatal myoclonus with pharyngeal, laryngeal, and lingual involvement may also be present (22). Seizures, peripheral neuropathy (that may be axonal, demyelinating or mixed), motor, or sensorimotor (24, 25) and *pes cavus* are also possible features (Figure 3). Later, with advancing age, other frequent findings can be observed, such as premature atherosclerosis, osteoporosis, and cardiovascular disease, which include ischemic heart disease, mitral valve insufficiency, abdominal aortic aneurysm, coronary artery dissection, and thickening of the interatrial septum due to lipomatous hypertrophy (1). There are also descriptions of cardiac autonomic dysfunction, ventricular tachycardia, and atrial fibrillation in the disease (7, 26). Osteoporosis represents a challenging chronic complication of CTX and commonly leads to important morbidity, especially in lately diagnosed patients and generally with poor response to CDCA therapy (1, 2, 6, 7). Childhood and juvenile-onset osteoporosis may be also a possible early complication of CTX and probably underdiagnosed during the first decades of life (1, 6).

Despite ataxia is usually considered the main gait disturbance presented by patients with CTX, pyramidal findings are more frequent than cerebellar signs (15) and cases of spinal xanthomatosis, characterized sometimes as pure forms of spastic paraparesis, have been reported in the literature (27–29). A recent literature review on spinal xanthomatosis, reviewed 34 cases, reporting a mean age of onset of the neurological symptoms of 24 years, with most cases presenting with complex hereditary spastic paraplegia (HSP) phenotype, presenting dementia, ataxia, polyneuropathy, seizures, and psychiatric disease as the complicating feature. Interestingly, 23.5% reported patients had spastic paraplegia as the sole neurological phenotype and only 31% of patients with spinal xanthomatosis presented xanthomas. On the other hand, cataracts and chronic diarrhea were frequent features being present in 78 and 65% of cases, respectively (30). Since the report by Burguez et al. (29) the center of one of the authors of the present manuscript (Saute JA) has been screening *CYP27A1* in the investigation of patients with HSP suspicion. Among 115 screened families, CTX was diagnosed in six of them, representing 5% of this cohort in southern Brazil (Saute JA personal communication), confirming that HSP phenotype should lead to CTX suspicion with biochemical or genetic screening for the disease.

The presence of two of four clinical hallmarks (premature cataracts, diarrhea, progressive neurologic signs, tendon xanthomas) should trigger comprehensive biochemical testing for CTX (31). Ophthalmologists may notice unexplained bilateral cataracts, which are a common symptom, especially in children and teenagers (32). Given that these are among of the early indications and symptoms, the association of juvenile cataracts and chronic diarrhea is particularly significant (33–35). Additionally, children and adolescents with psychiatric disorders including autism spectrum disorder, attention deficit hyperactivity disorder (ADHD), irritability, aggressive outbursts, or oppositional-defiant disorder should undergo further testing, especially in the context of consanguinity or in the presence of cataracts or chronic diarrhea (36).

In clinical practice, the Mignarri index of suspicion can be used to calculate the CTX prediction score and guide the best diagnostic approach for each individual. This index assigns different scores to certain groups of findings (Table 1): (i) family history, (ii) systemic signs, and (iii) neurological involvement (37). The highest indicator score (100) is attributable to “very strong indicator” (A), including positive family history of a sibling with CTX (A1), and/or presence of tendon xanthomas (A2). “Strong indicator” (B) with an individual score of 50 is given to the presence of consanguineous parents (B1), and/or juvenile-onset cataracts (B2), childhood-onset chronic diarrhea (B3), prolonged unexplained neonatal jaundice (B4), and/or ataxia or spastic paraparesis (B5), dentate nuclei signal changes at brain MRI (B6), and/or intellectual disability or psychiatric disturbances (B7). “Moderate indicator” (C) is attributable to individual score of 25 and given to early osteoporosis (C1), and/or epilepsy (C2), parkinsonism (C3), and polyneuropathy (C4) (7, 37, 38). Plasma cholestanol level assessment is indicated in patients with Mignarri scores ≥100. With previous high levels of plasma cholestanol or a Mignarri score ≥200 (including at least 1 “very strong indicator” or 4 “strong indicator”), there is a formal indication for genetic analysis of *CYP27A1* gene (2, 37, 39, 40). The clinical use of the Mignarri score should not limit the early investigation of patients with clinical features highly suggestive of CTX diagnosis (i.e., juvenile cataracts, childhood-onset chronic diarrhea), even in the absence of score values higher than 100 or 200 points or other clinical signs.

**Table 1:** Mignarri suspicion index for CTX [adapted and modified from (37)].

	**Very strong criteria (A)**	**Strong criteria (B)**	**Moderate criteria (C)**
Family history	Sibling with CTX	Consanguineous parents	–
Multisystemic involvement	Tendon xantomas	Juvenile cataract; childhood-onset chronic diarrhea; prolonged neonatal jaundice	Early osteoporosis
Neurological involvement	–	Cerebellar ataxia and/or spastic paraparesis; dentate nucleus signal change in neuroimaging; intellectual disability and/or neuropsychiatric involvement	Epilepsy; Parkinsonism; polyneuropathy

Score for each group of criteria: very strong criteria = 100/strong criteria = 50/moderate criteria = 25.

Obs. 1: Suspicion index scores of 200 or higher with at least one (A) criteria or four (B) criteria indicate the need of assessment of the *CYP27A1* gene analysis or, if unavailable, plasma cholestanol level evaluation.

Obs. 2: As diagnostic resources are limited in most centers, cases with suspicion index scores of 100 or higher should be evaluated initially with the assessment of plasma cholestanol levels. High plasma cholestanol levels indicate the need of further genetic analysis for CTX, as well as cases with normal plasma cholestanol levels but with a positive family history of a sibling with CTX also indicate the need of genetic testing.

Obs. 3: The Mignarri suspicion index can be used for patients with clinical suspicion at any age of symptom-onset, despite being more indicated for juvenile and adult patients.

As for the biochemical characteristics that provide subsidies to aid in the diagnosis, high serum concentrations of cholestanol are the main diagnostic marker of CTX (7). Elevation of other cholesterol precursors, such as 7-dehydrocholesterol and 8-dehydrocholesterol, is also commonly observed in plasma testing in CTX (41). High levels of bile alcohols, such as glucuronides, can be found in bile, plasma, and urine and are biomarkers for CTX. In tissues, cholesterol tends to be increased, while in plasma its concentration is normal or reduced. Other bile acid precursors in plasma and bile (such as lathosterol, lanosterol) are increased. Classical CTX form usually leads to significantly higher plasma levels of cholestanol than atypical forms and spinal CTX. Plasma levels of cholestanol and abnormal intermediates of bile acid synthesis may be elevated also in chronic cholestatic biliary tract diseases, such as Primary biliary cirrhosis and Progressive Familial Intrahepatic Cholestasis type 3 (*ABCB4*), and in inherited metabolic disorders, such as Niemann-Pick disease type C, sitosterolemia, familial hypercholesterolemia, and peroxisomal biogenesis disorders (1, 6, 42, 43). Drugs which promote abnormal activity of bile acid metabolism, such as intravenous propofol during total anesthesia, may lead to similar metabolic profiles to primary bile acid synthesis disorders (44). There is also important variation in plasma cholestanol levels in different ethnic and age groups, mainly comparing neonatal, childhood and adult (45). Chronic steroid use may reduce plasma cholestanol levels, leading to potential false-negative results and normal values (46), while hypothyroidism may lead to increased levels (1). In CSF analysis, it is possible to find high levels of cholestanol, cholesterol, fragments of apolipoprotein B, apolipoprotein-A1, and albumin (1, 7, 11). Liver biopsy may demonstrate the presence of electrodense deposits dispersed in the cytoplasm and crystal formation (1, 6, 39), although they are not routinely indicated. The quantification of the bile acid precursor 7 alpha-hydroxy-4-cholesten-3-one is being proposed as a rapid and potentially alternative diagnostic test for CTX (7, 12), as well as an optimal therapeutic biomarker during clinical follow-up (47).

Sequencing of *CYP27A1* should be performed in all patients with a suspected diagnosis of CTX. Some authors have suggested genetic testing in patients with high cholestanol levels or with very high clinical suspicion (34), but nowadays the accessibility of genetic testing is greater than dosing cholestanol for most centers. Bi-allelic pathogenic variants combined with typical clinical findings are diagnostic of CTX, but variants of unknown significance should always be confirmed with plasma cholestanol analysis (6). As genetic testing methods became more accessible and available for investigation in most centers specialized in Rare Diseases, organizing knowledge about potential clinic-genetic correlations in CTX has become a major challenge in clinical practice (Table 2). The development of specific diagnostic criteria for CTX became an essential measure for diagnostic purposes and future clinical trials (Table 3).

**Table 2:** Variants described in *CYP27A1* gene and the main clinical and genetic correlations associated with CTX (1, 2, 4–7, 9, 17, 18, 20, 29, 48).

**I. Variants associated with specific CTX phenotypes or features (1)**		
c.1214G>A (p.Arg405Gln)	Spinal CTX	
c.435G>T (p.Gly145Gly), c.1214G>A (p.Arg405Gln), c.1421G>A (p.Arg474Gln)	“Non-neurological” CTX: phenotype associated with marked multisystemic involvement and no clinical or neuroimaging evidence of neurological compromise.	
c.1421G>A (p.Arg474Gln)	Classical CTX	
c.646G>C (p.Ala216Pro)	Isolated neurological involvement (including adult-onset forms)	
c.1183C>T (p.Arg395Cys)	Acute neuropsychiatric disturbances and psychosis after CDCA discontinuation	
**II. Heterozygous carriers, polymorphisms, and disease risk associated with CYP27A1** **(****49****)**		
Increased susceptibility to cardiovascular disorders, gallstones, and sporadic amyotrophic lateral sclerosis (rs4674345)		
Some common polymorphisms in *CYP27A1* gene (rs4674345, rs4674338) are associated with premature aging, type 2 diabetes mellitus risk in obese population and higher cardiovascular risk.		
There are currently no other allelic disorders associated with *CYP27A1* pathogenic variants.		
**III. Population, founder effects and most prevalents variants** **(****1****)**		
Higher prevalence of CTX	Japanese; Italian (Sardinia); Sephardic Jewish from Moroccan origin; Druze ethnoreligious group in Israel	
Netherlands	c.1016C>T, c.1183C>T, c.1263+1G>A	
Italy	c.646G>C, c.1183C>T, c.1184+1G>A, c.1263+1G>A, deletion of 1.9 kb (including exons 7–9)	
Japan	c.1214G>A, c.1421G>A, c.1420C>T, c.435G>T	
Spain	Northwestern (c.1183C>T) and Southern (c.1213C>T)	
**IV. Specific features associated with the main observed variants in CYP27A1 gene** **(****1****)**		
No definite genotype-phenotype correlations have been established (1).		
c.1571T>G (p.Leu524Arg)	–	VUS (PP3, PM2); missense; homozygous; Turkey; high plasma cholestanol levels; late-onset spinal CTX. AF: absent in gnomAD aggregated.
c.1435C>T (p.Arg479Cys)	VCV000004254.17, rs72551322	Pathogenic (PP5, PM2, PM5, PM1, PP3); missense; compound heterozygous or homozygous; recurrent in Sardinia (Italy) and France; commonly childhood-onset, no diarrhea, no xanthomata; late-onset dementia and spastic paraparesis; may have normal plasma cholestanol levels. AF: 0.0043% (gnomAD aggregated).
c.1435C>G (p.Arg479Gly)	VCV000004267.7, rs72551322	Pathogenic (PP5, PM2, PM5, PM1, PP3); missense; compound heterozygous; Global (France, USA, Brazil). AF:0.0014% (gnomAD aggregated).
c.1421G>A (p.Arg474Gln)	VCV000004258.12, rs121908097	Pathogenic (PP5, PM2, PM5, PM1, PP3); missense, abnormal RNA splicing site change; compound heterozygous or homozygous; Brazil, Japan, USA, Netherlands; high plasma cholestanol levels. AF: 0.0016% (gnomAD aggregated).
c.1420C>T (p.Arg474Trp)	VCV000004259.14, rs121908098	Pathogenic (PP5, PM2, PM5, PM1, PP3); missense; compound heterozygous; Japan, Germany, USA, South Korea, Netherlands, Slovenia, China (Han population); high plasma cholestanol levels; late-onset pure spinal CTX, rapidly progressive. AF: 0.0024% (gnomAD aggregated).
c.1342_1343insCACC (p.Arg448fs*)	–	Pathogenic (PVS1, PM2, PP5); frameshift variant; compound heterozygous; Japan; late-onset disease, isolated bilateral Achilles tendon xanthoma, no neurological involvement (isolated neuroimaging features). AF: absent in gnomAD aggregated.
c.1263+1G>A (splice donor)	VCV000004262.14, rs397515355	Pathogenic (PVS1, PM2, PP5); canonical splice site variant, splice donor variant; compound heterozygous; China (Han population), France, USA, Japan; latent hotspot; systemic involvement not obligatory. AF: 0.0028% (gnomAD aggregated).
c.1214G>A (p.Arg405Gln)	VCV000004260.17, rs121908099	Pathogenic (PP5, PP3, PM2, PM5); missense; homozygous or compound heterozygous; South Korea, USA, Japan, France, China (Han population); AF: 0.004% (gnomAD aggregated).
c.1209C>G (p.Asn403Lys)	VCV000065837.2, rs587778781	VUS (PM2); missense; compound heterozygous; childhood-onset; no cataracts, no diarrhea, no xanthomata; AF: absent in gnomAD aggregated.
c.1198G>T (p.Val400Phe)	–	VUS (PM2, PP3); compound heterozygous; adult-onset; high plasma cholestanol levels; no significant systemic involvement (no cataracts, no diarrhea, no xanthomata). AF: absent in gnomAD aggregated.
c.1184+1G>A (splice donor)	VCV000065833.32, rs587778777	Pathogenic (PVS1, PM2, PP5); splice site mutation; compound heterozygous or homozygous; Iran, France; high plasma cholestanol levels; higher rates of non-syndromic intellectual disability; systemic involvement not obligatory. AF: 0.0163% (gnomAD aggregated).
c.1176_1177del (p.Glu392fs*)	–	Likely pathogenic (PVS1, PM2); compound heterozygous; Japan; high plasma cholestanol levels. AF: absent in gnomAD aggregated.
c.1169delT (p.Lys391fs*)	–	Likely pathogenic (PVS1, PM2); frameshift; homozygous; no cataracts; typical neuroimaging and neurological involvement. AF: absentin gnomAD aggregated.
c.1017G>C (p.Thr339Thr)	VCV0000284271.10, rs200553205	VUS (PP3, PM2, PP5); compound heterozygous; France; normal plasma cholestanol levels; pure neurological phenotype (late-onset dementia and spastic paraparesis, or childhood-onset disease); no diarrhea, no xanthomata. AF: absent in gnomAD aggregated.
c.1016C>T (p.Thr339Met)	VCV000004266.33, rs121908102	Pathogenic (PP5, PP3, PM2, PM5); missense; compound heterozygous; USA, Brazil, Germany, South Korea, Netherlands, China (Han population); high plasma cholestanol levels. AF: 0.0086% (gnomAD aggregated).
c.944_948delTGGCC (p.Leu315Glnfs*15)	VCV000004265.7, rs397515356	Pathogenic (PVS1, PM2, PP5); frameshift variant, premature translational stop signal; compound heterozygous; USA. AF: 0.002% (gnomAD aggregated).
c.850_854delinsCTC (p.Lys284fs)	–	Likely pathogenic (PVS1, PM2); homozygous; Italy; typical neurological disturbances without tendon xanthomata. AF: absent in gnomAD aggregated.
c.845-1G>A (acceptor site)	VCV000004256.12, rs397515353	Pathogenic (PVS1, PM2, PP5); canonical acceptor splice site, disruption of RNA splicing; homozygous or compoundheterozygous. AF: 0.0064% (gnomAD aggregated).
c.844+1G>A (splice donor)	VCV000004257.12, rs397515354	Pathogenic (PP5, PVS1, PM2); splice donor variant, aberrant RNA splicing; homozygous; Morrocan Jews, Germany, Netherlands, USA; spinal CTX, classical CTX. AF: 0.0011% (gnomAD aggregated).
c.784C>T (p.Arg262Cys)	VCV001284558.4, rs778371330	VUS (PM2); missense; compound heterozygous; late-onset spinal CTX, rapidly progressive; Japan, Netherlands, USA. AF: 0.0057% (gnomAD aggregated).
c.646G>C (p.Ala216Pro)	VCV000065885.20, rs201346271	Pathogenic (PP5, PM2, PP3); missense; compound heterozygous or homozygous; rare isolated neurological involvement. AF: 0.0032% (gnomAD aggregated).
c.526del (p.Asp176fs)	VCV000334367.12, rs765512351, rs886055630	Pathogenic (PVS1, PM2, PP5); frameshift variant, premature translational stop signal; homozygous; India (Eastern, Southern), Suriname, Netherlands, USA. AF: 0.0032% (gnomAD aggregated).
c.435G>T (p.Gly145Gly)	RCV000056114.6, rs58778796	Likely pathogenic (PP5, PM2); synonymous variant, alternative pre-mRNA splicing; compound heterozygous; USA, South Korea. AF: 0.0024% (gnomAD aggregated).
c.410G>A (p.Arg137Gln)	RCV000056176.9, rs587778818	Pathogenic (PP5, PM2, PM5, PP3, PM1); missense; homozygous cases with increased plasma cholestanol levels; compound heterozygous cases with mild CTX phenotype, spinal CTX and mild lab changes (including normal plasma cholestanol levels). AF: 0.0039% (gnomAD aggregated).

AF, allele frequency; VUS, variant of uncertain significance.

Obs. 1: AF presented in this table according to gnomAD v2.1.1 (*CYP27A1*: ID ENSG00000135929.4). Currently, 118 ClinVar variants in *CYP27A1* gene are classified as pathogenic or likely pathogenic (and only 48 of them are included in gnomAD, up to 30th October, 2022).

Obs. 2: All variants presented in this table consider NM_000784.4 (*CYP27A1*) as the reference primary transcript.

Obs. 3: There are no established correlations involving specific variants and different levels of biomarkers in primary newborn screening (50).

Obs. 4: Most typical pathogenic variants involve the adrenodoxin-binding (exon 6) and heme-binding (exons 8 and 9) domains of *CYP27A1*.

Obs. 5: Other rare variants found in single descriptions are not summarized in this table (eg., c.255+1G>T, c.256-1G>T, c.379C>T, c.1561dupA, c.1537C>T, c.1264A>G, c.435-12G>T, c.434G>A, c.850A>T).

Obs. 6: Pathogenicity classification of each variant presented in the table takes into consideration the 28 criteria of the American College of Medical Genetics and Genomics (ACMG) Standards and Guidelines, 2015.

**Table 3:** Diagnostic criteria and categories for CTX, based on Sekijima's (17) and modified from Stelten's criteria (6).

**Diagnostic category:**
(i) Definite CTX: One or more symptoms or signs in criteria (A) + criteria B ±C ±D (ii) Probable CTX: One or more symptoms or signs in criteria (A) + criteria B ±D (iii) Possible CTX: One or more symptoms or signs in criteria (A) + criteria B
Criteria (A): Signs and symptoms (seven different classes): juvenile or adult-onset tendon xanthoma; intellectual disability or progressive neurological or neuropsychiatric involvement (e.g., cognitive decline, spastic paraparesis, cerebellar ataxia, polyneuropathy, seizures, parkinsonism, dystonia); childhood or juvenile-onset cataracts; juvenile coronary artery disease; chronic unexplained childhood-onset diarrhea; juvenile osteoporosis; prolonged neonatal cholestasis.
Criteria (B): Biochemical studies: raised levels of plasma/serum cholestanol levels.
Criteria (C): Genetic testing studies: presence of biallelic pathogenic variants in the *CYP27A1* gene (homozygous or compound heterozygous).
Criteria (D): Exclusion of other potential differential diagnosis: especially for abnormal plasma cholestanol levels and atypical profile of bile alcohols (e.g., sitosterolemia, hypothyroidism, obstructive biliary tract disease, familial hypercholesterolemia)

Obs. 1: Despite the existence of criteria B, as discussed in other topic of this review manuscript, there are several cases of genetically-proven CTX with normal plasma cholestanol levels during assessment.

Obs. 2: Presentations with pure behavioral involvement or affective/mood disorders are generally not included as a single positive neurological sign in criteria A.

Obs. 3: The absence of signal changes involving the dentate nuclei and cerebellar and deep and periventricular white matter on brain MRI studies does not rule out the diagnosis of CTX, as well as normal spine MRI studies do not exclude the diagnosis of spinal CTX.

Obs. 4: Identification of biallelic pathogenic variants may be performed by single-gene sequencing analysis, by next-generation sequencing-based multigene panels, by large-scale sequencing techniques (whole-exome or genome sequencing) or rarely by deletion/duplication analysis by MLPA (multiplex ligation-dependent probe amplification) or quantitative PCR techniques.

Regarding differential diagnoses, sitosterolemia, familial hypercholesterolemia (both of which can also manifest with tendon xanthomas), Smith-Lemli-Opitz syndrome (characterized by elevated 7-dehydrocholesterol, which may also be present in some CTX patients), other inborn errors of bile acid metabolism (such as HSP type 5A), and non-specific liver disease are among the disorders with features similar to CTX (40, 50–52). Progressive neurologic symptoms, as well as cataracts and chronic diarrhea, can distinguish CTX from these disorders (34, 50). Congenital diarrhea and Alagille syndrome are important differential diagnoses in childhood onset, as well as other causes of neonatal jaundice. In adult patients, differential diagnosis is made with other causes of progressive neurologic disease, such as HSP, hereditary cerebellar ataxias, multiple sclerosis, leukodystrophies, mitochondrial disease, histiocytosis, and other causes of acquired ataxia, and in these cases tendon xanthomas and cataracts are among the most important clues for CTX (53).

The considerable diagnostic delay before the correct diagnosis and proper treatment might be prevented if CTX was included in national newborn screening programs. Newborn screening with dried blood spots is considered by several groups a key step for early diagnosis and treatment (54). However, inclusion in newborn screening programs should be done cautiously and initially in a research context due to the likelihood of detecting mild variants that may remain asymptomatic for a longer period without treatment (6). There is not a definite metabolite or battery tier to perform metabolic or genetic neonatal screening for CTX. However, the most characteristic biomarker in CTX positive newborns after screening was 5-β-cholestane-3α,7α,12α,25-tetrol,3-*O*-β-D-glucuronide (GlcA-tetrol) (54, 55), and both GlcA-tetrol and the ratio of GlcA-tetrol to tauro-chenodeoxycholic acid from dried blood spots represent the most accurate diagnostic biomarkers for newborn screening (6, 55, 56).

## 4. Neuroimaging findings

The typical neuroimaging finding of CTX is T2-weighted (T2W) hyperintensity in the dentate nucleus, with cerebellar hypointensity occasionally being seen in the late stage as a result of hemosiderin depositions and microhemorrhages following cerebellar vacuolation (57).

**Figure 4:**
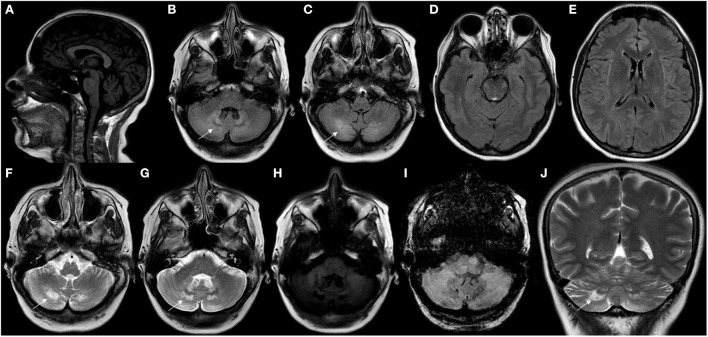
Neuroimaging findings in CTX. **(A)** Sagittal T1W brain MR imaging (MRI) disclosed mild cerebellar atrophy. **(B–G)** Axial brain MRI showed hyperintensity involving cerebellar white matter and dentate nuclei (white arrows) in FLAIR **(B–E)** and T2W imaging **(F, G)**. Axial brain MRI showing mild hypointensity involving cerebellar white matter and dentate nuclei in T1W **(H)** and SWI sequences **(I)**. Coronal brain MRI showing hyperintensity (white arrow) involving cerebellar white matter and dentate nuclei in T2W imaging **(J)**.

**Figure 5:**
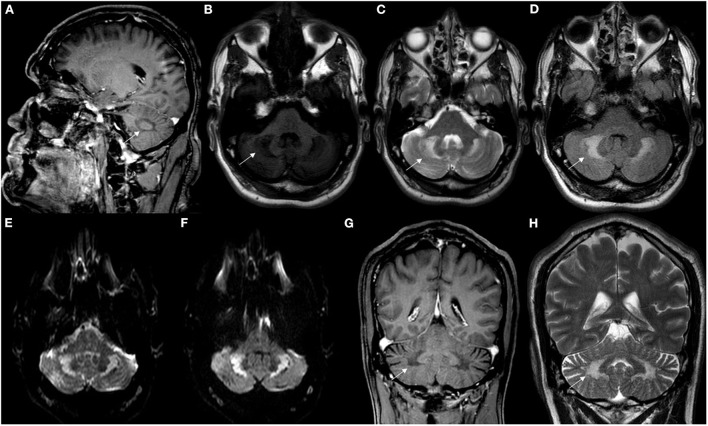
Neuroimaging patterns observed in CTX. **(A)** Sagittal T1-weighted brain MRI showed hypointense signal in deep cerebellar white matter (white arrow). Axial brain MRI disclosed signal change in deep cerebellar white matter (white arrows) hypointense in T1W **(B)** and hyperintense in T2W **(C)** and FLAIR sequences **(D)**. Axial brain MRI disclosed hyperintensity in deep cerebellar white matter in DWI **(E)** and ADC sequences **(F)**. Coronal brain MRI showed hypointensity in the deep white matter in T1W imaging **(G)**, as well as corresponding hyperintensity (white arrows) in T2W imaging **(H)**.

A review article of 38 patients has shown brain MRI abnormalities in 84% of patients, with supra and infratentorial cortical atrophy, subcortical and periventricular white matter abnormalities, brainstem lesions, cerebellar atrophy and other cerebellar parenchymal abnormalities involving the dentate nuclei and the surrounding white matter as the main findings (9). T2W and FLAIR brain MRI may show symmetric hyperintense lesions in the periventricular white matter, posterior limbs of internal capsules, globus pallidum, cerebral peduncles extending into the substantia nigra, anterior region of the pons, inferior olive, or in the cerebellar parenchyma, involving the dentate nuclei and the surrounding white matter, which were hypointense on T1W and diffusion-weighted images (DWI) (7, 40) (Figures 4, 5).

The dentate nuclei may present with hypointensities on T2W/FLAIR and susceptibility-weighted (SW) images over time. T2W/FLAIR signal abnormalities in the dentate nuclei were the most common findings in patients with CTX (30). The reasons for preferential involvement of the dentate nucleus remain unclear (12, 30). A significant clinical-imaging correlation was only found between the extent of dentate hyperintense lesions and disability expressed by the modified Rankin Scale (30).

The distribution of lesions along corticospinal tracts or in the cerebellum were consistent with the clinical presentation of pyramidal or cerebellar signs, whereas abnormalities of the substantia nigra may be associated with Parkinsonian features (30, 40).

**Figure 6:**
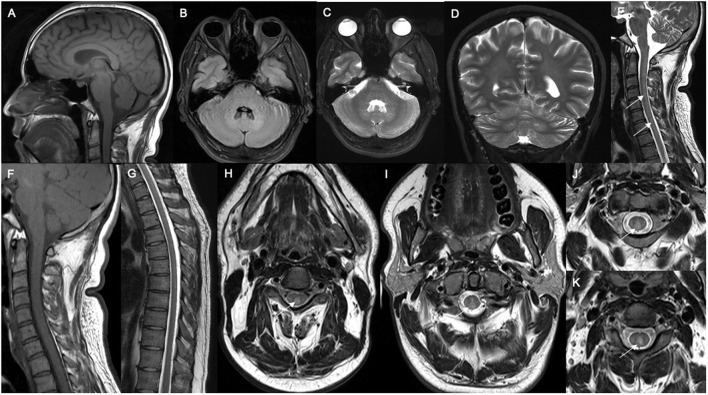
Neuroradiological features in spinal CTX. **(A)** Sagittal brain MRI showed normal cerebellar and corpus callosum structure. Axial brain MRI showed mild hyperintensity in dentate nucleus in FLAIR **(B)** and T2W sequences **(C)**, as well as hyperintensity in dentate nucleus in coronal T2W imaging **(D)**. Sagittal spine MRI showed hyperintense (white arrows) in T2W imaging **(E)** and isointense change in the dorsal column of the cervical spinal cord in T1-weighted imaging **(F)**. Sagittal thoracic spine MRI showed normal structure **(G)**. Axial cervical spine MRI showed marked hyperintensity of the dorsal column (white arrows) in T1W **(H)** and T2W imaging **(I–K)**.

Spinal xanthomatosis may present with non-enhancing long T2W hyperintense lesions predominantly involving the central and posterior cord (Figure 6). One study has found it to have a relatively mild clinical course, compared with the classic form of the disease (30). This case series with 33 patients reported that patients usually presented pyramidal signs and 48% had dorsal column signs. One of the patients presented with late-diagnosed CTX and after treatment discontinuation had psychiatric symptoms and marked spinal xanthomatosis (rare), which manifested as spastic paraparesis in the absence of xanthomas. Spinal MRI revealed new linear hyperintensities of the corticospinal and gracile tracts (30, 57).

Neuroimaging findings, despite being highly variable among patients, generally disclose features which are rarely reversible in multi-imaging modalities after treatment with CDCA, even in individuals with early diagnosis of CTX (13). There is not, however, a direct correlation between the severity of neurological compromise and the extension of dentate nuclei and white matter involvement, including patients with severe motor compromise and cognitive decline with unremarkable neuroimaging studies. Neuroimaging features are quite similar in both adult and childhood-onset cases, despite a more typical pattern being identified in adult patients (1, 13, 57).

In summary, brain MRI shows diffuse cerebellar atrophy; ex-vacuum dilatation of the IV ventricle; symmetric hyperintensity of the dentate nuclei and of the cerebellar white matter with some associated DWI hypointensities in the adjacent zone; a soft hyperintensity on T2W and FLAIR may be noted in the superior cerebellar peduncles and in the pyramidal tracts; initial signs of cerebral atrophy may also present be with enlargement of both the insular and frontal spaces (13).

Additionally, magnetic resonance spectroscopy (MRS) may reveal typical lipid peaks, increased choline, and decreased *N*-acetyl-aspartate peaks in the involved regions, which indicates extensive axonal damage and mitochondrial dysfunction (40). Functional dopaminergic studies showed presynaptic denervation, which is consistent with the mild improvement with levodopa in some patients (58, 59).

Usually tendon xanthomas appear as hypo- to isointense on T1W images and showed low to intermediate signal on T2W images. Bilateral Achilles tendons were most frequently involved. CT scans may show soft tissue enlargement with areas of low attenuation. This may be related to abnormal lipid deposition (13, 38, 57).

## 5. Clinical management and therapeutic approaches

Generally, neurologic and neuropsychologic evaluations, plasma cholestanol concentration, brain MRI, echocardiography, and total-body bone density should be evaluated annually; more frequent surveillance may be indicated in newly diagnosed patients until biochemical indicators of disease stabilize. Of note, because cholestanol levels may require considerable time to return to a normal range (reduction of 91 μmol/l in an average follow-up of 34 months) after specific treatment initiation, other substrates in the cholestanol pathways, such as intermediate bile alcohols, could be used for short-term follow-up or as surrogate outcomes in clinical trials (7).

In CTX pathophysiology, there is markedly reduced production of bile acids, especially chenodeoxycholic acid (CDCA), and to a lesser extent cholic acid (CA). As CDCA and CA have a negative physiologically feedback on 7-α-hydroxylase, the rate-limiting enzyme of bile acids synthetic pathway, in CTX 7-α-hydroxylase activity is highly enhanced (60). This results in reduced synthesis of CDCA, high production of cholestanol and its subsequent accumulation in different tissues, as well as normal or low levels of cholestanol in plasma and bile alcohols in urine (61). Evidence that cholestanol may be neurotoxic is supported by the finding of cholestanol deposition and apoptosis in neuronal cells, most notably Purkinje cells, in the cerebellum of rats fed a 1% cholestanol diet (62). CDCA also blocks and antagonizes GABA_A_ and NMDA receptors (63) (Figure 1).

Given the reduced synthesis of CDCA and high production of cholestanol, as well as the recent evidence that cholestanol may be neurotoxic, CDCA has become the standard of care for CTX patients. It prevents the accumulation of cholestanol by inhibiting bile acid synthesis through a negative feedback pathway (31). This drastically lowers plasma cholestanol concentrations in patients and its accumulation in tissues. While initial studies with CDCA reported clear short-term clinical benefit in most patients with CTX, long-term studies have rather reported stabilization in some patients (61). In 1984, one of the first studies was published that spoke in favor of long-term benefit from CDCA therapy in outcomes such as decreased serum cholestanol, improved neurological examination and electroencephalographic findings (16). Despite this, there have been no specific randomized placebo-controlled clinical trials of CDCA in patients with CTX to this date.

CDCA treatment typically does not significantly reduce tendon xanthomas or improve cataracts, but can stabilize or improve neurologic manifestations, including cognitive deterioration, pyramidal tract signs, and cerebellar deficits (15). Therefore, given the natural course of CTX, the primary aim of treatment is stabilization or improvement of neurological signs and symptoms based on results of retrospective trials (31).

While most CTX patients do well in response to CDCA therapy, others continue to deteriorate neurologically, especially patients diagnosed over the age of 25 years who already have significant neurologic disease (64). When significant neurologic pathology has occurred, the effect of treatment seems to be limited (15). Early diagnosis and treatment in CTX are imperative to prevent potentially irreversible neurological damage and it changes the disease course in a positive way, alleviating both the neurologic and systemic symptoms of CTX. In a study with 43 CTX patients with a follow-up of 8 years, cognitive impairment (74%), premature cataracts (70%), tendon xanthomas (77%), and neurologic disease (81%) were the most frequent conditions, and treatment with CDCA improved symptoms in 57% of patients, despite of 20% continued to deteriorate (64).

In the largest retrospective cohort study of CDCA treatment with 56 patients, all patients diagnosed and treated before the age of 24 had complete resolution of previous neurologic symptoms and no new onset symptoms, while 61% of patients diagnosed and treated after the age of 24 had neurologic deterioration, with parkinsonism as the main treatment resistant feature (6). These findings suggest that CDCA treatment should be instituted as soon as possible, and that early diagnosis is paramount to good outcomes in this disease (65).

The currently recommended dosing for CDCA ranges from 5 to 15 mg/kg per day in children and 750 mg per day, in three divided doses, in adults. There is a formal recommendation of a slowly progressive dosing introduction with 500 mg per day, for 2 weeks, followed by a weekly increase of 250 mg per day, until the recommended dose is reached (6). If serum cholestanol or urine bile alcohols remain elevated after 3 months, CDCA may be raised up to 1,000 mg per day. For children and adolescents, it is recommended an initial dose of 5 mg/kg per day, in three divided doses. Few specific adverse events or safety concerns have been reported for CTX patients treated with CDCA, with most reports indicating no major adverse events. Discontinuation of therapy due to adverse events occurs in <5% of cases (31). Hepatotoxicity is considered the major concern with CDCA, however in most cases with minor serum aminotransferase elevations (66). Hepatotoxicity leads to the need of dosing adjustment (67), despite in most cases minor serum transaminase elevations (up to three times the upper limit of normality) represent a transient phenomenon with complete resolution in up to 6 months after drug discontinuation. Patients with serum aminotransferase levels over three times the upper limit of normality and evolving with recurrence of such laboratory changes after reintroduction of CDCA may discontinue therapy. CDCA restart is recommended generally at lower initial doses of 5 mg/kg per day and maintained at such dosing with no significant complications (31). If patients evolve with persistent diarrhea or severe gastrointestinal complaints, transient reduction of the recommended dosing is performed until improvement of symptoms, when effective dosage is restarted. CDCA therapy is contraindicated in patients with moderate to severe hepatocyte dysfunction, intrahepatic cholestasis, primary biliary cirrhosis, sclerosing cholangitis, biliary pancreatitis, biliary gastrointestinal fistula, acute cholecystitis or cholangitis, or biliary tract obstruction (31, 64). Patients with absolute contraindication to CDCA use or severe adverse events may potentially benefit from the alternative use of CA (68).

Early reports of combination therapy with low doses of CDCA with HMG-CoA reductase inhibitor pravastatin suggested that this combination reduced plasma levels of cholestanol and avoided the increase in triglyceride and low-density lipoprotein (LDL)-cholesterol with CDCA alone; however, the follow-up period was too short to detect relevant clinical changes (69). Other case reports also favored the combination of CDCA with HMG-CoA reductase inhibitors like simvastatin and atorvastatin, with biochemical response and reports of improvements of peripheral neuropathy and cognitive symptoms when statin was added to CDCA (70, 71). LDL-apheresis with CDCA and HMG-CoA reductase inhibitor is another possible approach, despite the consistent reduction of cholestanol to normal or even subnormal levels, a definite improvement of clinical symptoms was not noted with this aggressive cholestanol lowering therapy (72, 73). CDCA alone presents stronger evidence on clinical outcomes than combined therapy with CDCA and HMG-CoA reductase inhibitor. In a recent consensus statement with Delphi method, the expert panelists considered CDCA alone the preferred first line therapy for CTX, but also considered that combination therapy with HMG-CoA reductase inhibitor improves/stabilizes the prognosis. The panel disagreed that LDL apheresis improves/stabilizes prognosis (6).

Previous studies have also evaluated the possible role of CA therapy in the management of patients with CTX with variable patterns of clinical response, especially regarding neurological involvement (49, 74). Through the suppression of endogenous bile acid biosynthesis by negative feedback mechanisms, similar to CDCA, CA supplementation provided significant reduction in the urinary excretion and serum production of intermediate biomarkers of bile acid biosynthesis pathway (75). A retrospective Franco-Belgian multicentric study evaluated the safety and efficacy of CA in the treatment of both CDCA-naïve and non-naïve patients with CTX (60). More than 80% of individuals had clinical improvement or stabilization and marked reduction of plasma cholestanol levels during treatment period, disclosing a possible role of CA as a second-line or alternative therapy in patients who presented moderate to severe side effects with CDCA therapy (60). There is currently no consensus regarding the use of CA as a monotherapy in CTX (6). Other studies have evaluated the potential role of several other compounds, such as cholestyramine (76), hydrophilic ursodeoxycholic acid (76), ursodeoxycholic acid (76) and, in biochemical and clinical parameters of patients with CTX, however no significant responses or benefits were observed (76).

Finally, preclinical studies showed that single intravenous administration of adeno-associated virus (AAV) expressing *CYP27A1* directed to liver provided full metabolic restoration of the disease in a transgenic mice model of CTX in a greater extent than CDCA (77), being a promising therapeutic option that should be pursued by future studies.

## 6. Prognosis

Age at definite diagnosis and treatment introduction as early as possible represents the most important prognostic factors related to treatment responses and outcomes, especially in asymptomatic patients or individuals without significant neuropsychiatric involvement (1, 6, 7). Progressive reduction of plasma cholestanol levels after treatment initiation leads to slowing clinical progression of CTX (6). Patients with typical brain MRI involvement of the deep cerebellar white matter with vacuolation seen as hypointense on T1-weighted and FLAIR sequences generally evolve with worse prognosis (60), as well as the absence of dentate nuclei signal changes is generally associated with better prognosis (6). Some *CYP27A1* pathogenic variants have been also associated with more severe neurological and multisystemic involvement and then worse prognosis (Table 2). As CDCA represents a specific drug therapy developed for continuous use, long-term treatment availability for initiation and maintenance represents a key issue for better neurological and systemic outcomes and increased life expectancy (6).

## 7. Conclusion

CTX is a rare and potentially treatable genetic disease that results in multisystemic involvement. The main cause of disability results from neurologic manifestations including pyramidal signs, ataxia, and cognitive impairment. Neuroimaging with typical T2W hyperintensity in the dentate nucleus, bilateral juvenile cataracts and the presence of tendon xanthomas are important clues for diagnosis. Treatment with CDCA is safe and appears to be effective based on results of retrospective studies, especially if initiated early. Prompt diagnosis, possibly with neonatal screening, may significantly reduce the burden of this disease.

## Author contributions

PN and AB: conception and design, acquisition of data, analysis and interpretation of data, writing of the first draft, review and critique, and final approval of the version. RR, SV, DA, VG, HF, CS, DD, AP, and WP: conception and design, analysis and interpretation of data, writing of the first draft, review and critique, and final approval of the version. JS, PS, and PB-N: conception and design, acquisition of data, writing of the first draft, review and critique, and final approval of the version. All authors contributed to the article and approved the submitted version.
